# Comparison of classification models for adolescent’s emotional and behavioral problems

**DOI:** 10.1093/jamiaopen/ooag144

**Published:** 2026-07-30

**Authors:** Akhyt Tilyeubai, Javzmaa Tsend, Nyamdavaa Uugandavaa, Bayarmaa Vaanchindorj, Purevdolgor Luvsantseren, Ajnai Luvsan-Ish, Baasandorj Chilkhaasuren, Galbadrakh Chuluunbaatar, Enkh-Urel Enkhjargal

**Affiliations:** Department of Physics & Medical Informatics, School of Biomedicine, Mongolian National University of Medical Sciences, Ulaanbaatar, 14210, Mongolia; Department of Physics & Medical Informatics, School of Biomedicine, Mongolian National University of Medical Sciences, Ulaanbaatar, 14210, Mongolia; Department of Physics & Medical Informatics, School of Biomedicine, Mongolian National University of Medical Sciences, Ulaanbaatar, 14210, Mongolia; National Center of Mental Health, Ulaanbaatar, 13340, Mongolia; Department of Physics & Medical Informatics, School of Biomedicine, Mongolian National University of Medical Sciences, Ulaanbaatar, 14210, Mongolia; Department of Physics & Medical Informatics, School of Biomedicine, Mongolian National University of Medical Sciences, Ulaanbaatar, 14210, Mongolia; Department of Physics & Medical Informatics, School of Biomedicine, Mongolian National University of Medical Sciences, Ulaanbaatar, 14210, Mongolia; Department of Physics & Medical Informatics, School of Biomedicine, Mongolian National University of Medical Sciences, Ulaanbaatar, 14210, Mongolia; Department of Physics & Medical Informatics, School of Biomedicine, Mongolian National University of Medical Sciences, Ulaanbaatar, 14210, Mongolia

**Keywords:** data mining, accuracy, specificity, sensitivity, measure, statistical tests

## Abstract

**Objectives:**

Mental disorders are very common among children and adolescents around the world. In Mongolia, the international Strengths and Difficulties Questionnaire (SDQ) is used to detect mental disorders in adolescents. In this article, different types of classification methods were compared to determine adolescent emotional and behavioral problems using the SDQ.

**Materials and Methods:**

Data were collected from teenagers, teachers, and parents in Govi-Altai Province, and the databases were created for each group. The teenager database was divided into 10 folds using cross-validation, and the models were developed using classification methods and evaluated using performance measures. The results were mainly analyzed using the Bayes model.

**Results:**

The teenagers have emotional and behavioral problems due to emotional and peer interactions, but they were at risk of developing disorders due to hyperactivity and behavioral changes.

**Conclusion:**

Future work will examine the difficulties encountered in the creation of emotional and behavioral problems among the teenagers involved in the study using a progressive classification method.

## 1. Introduction

During adolescence, youths undergo major biological and psychosocial changes and show distinct protective responses to stress.^1^^,^^2^ Worldwide, an estimated 13% of 10-19 years have a mental disorder. Because poverty, abuse, and violence heighten risk, reducing adversity, fostering emotional well-being, and ensuring access to mental health care are essential.^3^^,^^4^ In Mongolia, 1 289 587 children were under the age of 19 in 2020, a 0.9% rise since 2015. A 2013 study found that 60.5% were healthy, 30.5% had emotional or behavioral problems, and 9% had disorders; frequent issues were heavy gaming (73.8%), lying (50%), stealing (48.8%), and fighting (41.3%). Emotional and physical symptoms were common, such as restlessness (47.5%), depression (43.8%), anxiety (38.8%), and headaches (15.0%). Provincial surveys showed that 58.6% were healthy, 36.1% had problems, and 5.2% had disorders in Govi-Altai (2018-2019), and that 41.8%, 51.3%, and 6.9% in Umnugovi.^1^^,^^2^^,^^4^

The SDQ questionnaire is widely used to identify mental health problems in adolescents. In collaboration with health researchers, we will develop classifiers to predict emotional and behavioral problems using data-mining methods such as C5.0, Naïve Bayes, and JRip. C5.0 often yields high accuracy, supports cost-sensitive learning, and provides interpretable trees, but it can overfit and is parameter sensitive.^5^^,^^6^ Naïve Bayes is fast and stable on medium-sized data but is limited by its independence assumptions.^7^^,^^8^ JRip produces readable rules and tolerates noise yet may lag in accuracy and is pruning-sensitive.^8^ This research aligns with Mongolia’s science, technology, and innovation priorities in AI, big data, and product development and advances national medical science, focusing on child and adolescent mental health.

## 2. Materials and methods

### 2-1. Classification

The Strengths and Difficulties Questionnaire (SDQ) measures adolescent mental health and has identical parent, teacher, and self-report forms. It scores Emotional (3, 8, 13, 16, 24), Conduct (5, 7, 12, 18, 22), Hyperactivity/Inattention (2, 10, 15, 21, 25), and Peer (6, 11, 14, 19, 23) problems; items are scored 0-2, and the total score ranges from 0 to 40, with norms of 0-15 normal, 16-19 borderline, and 20-40 abnormal.^9^

As the data from the SDQ are categorical, we considered classification methods. Classification involves 2 steps. In the learning step, we will develop the models using a training set with predefined attributes and class labels.^10^

C5.0 is a decision tree algorithm that builds a model by repeatedly splitting a dataset based on the field with the highest information gain. This process continues until the sub-samples can no longer be split. After reaching the lowest level of splits, the model is pruned to remove those that do not significantly add value. C5.0 utilizes entropy to assess the purity of the splits, where a value of 0 indicates complete homogeneity and a value of 1 indicates maximum impurity.^11–14^

Bayesian classification, based in Bayes’ theorem, performs comparably to decision tree and certain neural network classifiers, demonstrating high accuracy and speed, especially with large databases.^10^^,^^15^^,^^16^

A rule-based classifier uses IF-THEN rules for classification, where the “IF” part (antecedent) contains conditions that are logically combined using AND, and the “THEN” part (consequent) indicates the classification outcome. The rule coverage is the proportion of data tuples that contains the rule, and its accuracy is determined by the percentage of correctly classified tuples. Rules can be extracted from training data using sequential covering algorithms, which learn rules one at a time, with the goal of covering many tuples of a specific class. Examples of these algorithms include AQ, CN2, and RIPPER.^10^

### 2-2. Model evaluation and selection

We used different methods to build more than one classifier in the first step and evaluated the models’ performance using metrics such as accuracy, sensitivity, and specificity in the second step.^10^

#### 2-2-1. Metrics for evaluating classifier performance

In the second step, measures such as accuracy and sensitivity are computed based on the confusion matrix that predicted the positive sets (TP) and negative sets (TN) correctly classified by the classifier, as well as the negative sets (FP) and positive sets (FN) incorrectly classified by the classifier. Then, the models are evaluated. Accuracy is the proportion of the test dataset that is correctly classified by the classifier. When the distribution of the data set shows a significant majority of negative class and a minority positive class, measures of sensitivity and specificity are also considered. Sensitivity refers to the proportion of positive tuples that are correctly identified, while specificity refers to the proportion of negative tuples that are correctly identified.

#### 2-2-2. Cross-validation

The study employed cross-validation, specifically with k = 10, to evaluate the classifiers. The data were divided into 10 folds, and the model was trained and tested 10 times, with each fold serving as the test set once. The accuracy was determined by counting the correct classifications across all iterations and dividing by the total number of data points.^10^

#### 2-2-3. Statistical tests of significance

To determine whether there was a difference in the mean error rates between these models, we used a paired-samples *t*-test that expresses statements “One model is better than the other by a margin of error of ± 4%”.^10^^,^^16^ For every *i*th round of 10-fold cross-validation, the error rates ($\mathrm{err}{\left(M_{1}\right)}_{i}$, $\mathrm{err}{\left(M_{2}\right)}_{i}$) of the M_1_ and M_2_ models are computed. These error rates were averaged to obtain the mean error rates ($\bar{\mathrm{err}}{(M}_{1}),\;\bar{\mathrm{err}}{(M}_{2})$) of M_1_ and M_2_, and the variance of these mean error rates was calculated. Then, the *t*-statistic is computed with *k*−1 degrees of freedom for k samples. In our study, the *k* samples were 10 error rates obtained from 10-fold cross-validations for each model. The *t*-statistic for pairwise comparison was computed as follows:


(1) $$t=\frac{\bar{\mathrm{err}}{(M}_{1})\;-\;\bar{\mathrm{err}}(M_{2})\;}{\sqrt{var(M_{1}-M_{2})/k}},$$

(2) $$\mathrm{var}(M_{1}-M_{2})=\frac{1}{k}\sum_{i=1}^{k}{\left[\mathrm{err}{\left(M_{1}\right)}_{i}-\mathrm{err}{\left(M_{2}\right)}_{i}-\bar{(\mathrm{err}}{(M}_{1})\;-\;\bar{\mathrm{err}}(M_{2}))\right]}^{2}.$$

Let $\mathrm{err}{\left(M_{1}\right)}_{i}$ be the error rate of model $M_{1}$ in round I and let $\mathrm{err}{\left(M_{2}\right)}_{i}$ be the error rate of model $M_{2}$ in round *i*.

Let $\bar{(\mathrm{err}}{(M}_{1})$ be the mean error rate for $M_{1}$, Let $\bar{(\mathrm{err}}{(M}_{2})\;$be the mean error rate for $M_{2}$.

The goal is to determine whether there is a significant difference between M_1_ and M_2_ at the 95% of the population by using the *t*-statistic. We look up the *t*-value corresponding to k−1 degrees of freedom, specifically at 0.025. If the calculated *t*-value is greater than z or less than −z, it falls in the rejection region, allowing us to reject the null hypothesis. This indicates that the means of M1 and M2 are significantly different.

## 3. Experimental results

Resolution No. 262 of the Medical Ethics Committee of Mongolia, dated January 20, 2022, granted ethical approval for the research titled “Investigation of the Mental Health Trends of Adolescents.”

In cooperation with the National Center of Medical Health (NCMH), we collected data using SDQ from 3764 teenagers, parents/guardians, and classroom teachers in Govi-Altai province. Based on a cross-sectional study conducted among the adolescents of the province, with a confidence level of 98%, an error margin of 2%, a population proportion of 50%, and a population size of 10 241, we can confidently state that a sample size of 3091 is sufficient to represent the population. The population size is the adolescents of Govi-Altai province.

**Figure 1.**
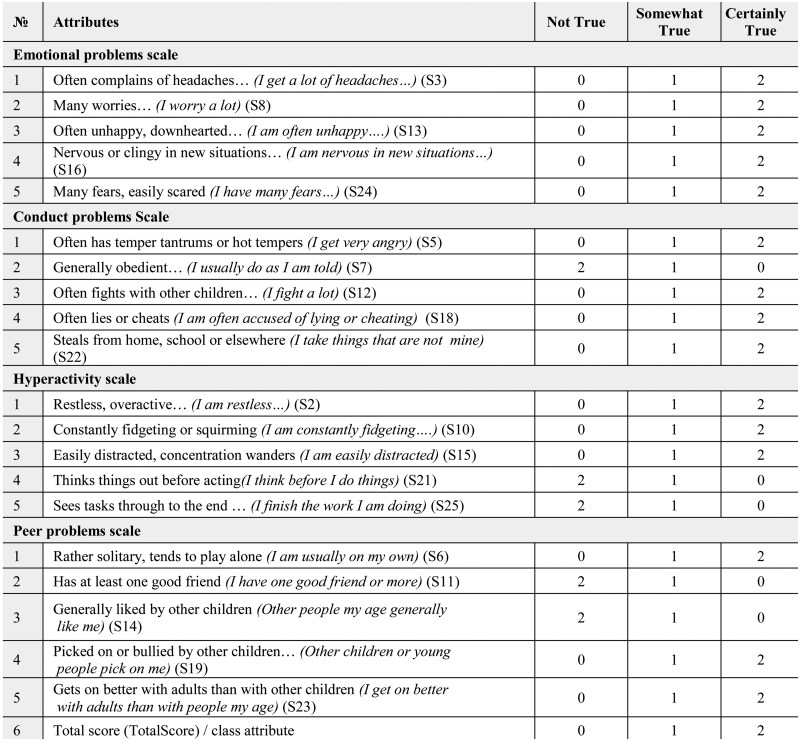
Scoring symptom of SDQ for the teenager database.

Figure 1 shows the emotional, behavioral, hyperactivity, and peer relationship questionnaires in the SDQ.

Table 1 shows the normal, borderline, and abnormal state ratings for the sum of the SDQ’s emotional, behavioral, inattention/hyperactivity, and peer relationship indicators. It will also be evaluated for each group.

**Table 1.** SDQ scoring value.

SDQ scoring	Normal (0)	Borderline (1)	Abnormal (2)
Total = E + B + H + P	0-15	16-19	20-40
Emotional symptoms E = (S3 + S8 + S13 + S16 + S24)	0-5	6	7-10
Behavioral problems B = (S5 + S7 + S12 + S18 + S22)	0-3	4	5-10
Hyperactivity H = (S2 + S10 + S15 + S21 + S25)	0-5	6	7-10
Peer problems P = (S6 + S11 + S14 + S19 + S23)	0-3	4-5	6-10

The total score of 0-40 is calculated based on the sum of indicators of adolescent emotions, behavior, inattention/hyperactivity, and peer relationships. If the total score of a teenager is 0-15, it means that the teenager is normal or healthy; when the total score is 16-19, it indicates that the teenager has a borderline state or emotional and behavioral problems; when the total score is 20-40, it indicates an abnormal state or emotional and behavioral disorders.

**Figure 2.**
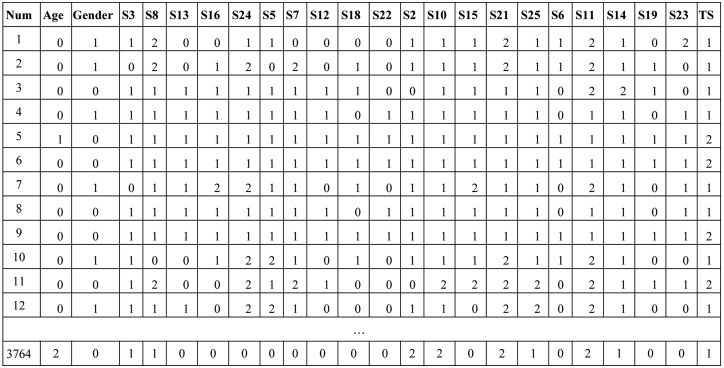
The teenager database.

We created a teenager evaluation database by calculating the general emotional and behavioral state of 3764 adolescents. The database has 3764 rows and 55 attributes, including general information, demographics, emotional and behavioral indicators, total score, difficulties, and influencing factors. From this, 23 attributes, such as the gender and age of adolescents, their emotional and behavioral characteristics, and total score, were selected, and the algorithms were tested. The total score (TS) was the class attribute of the dataset (Figure 2).

There were 1070 borderline, 881 abnormal, and 1813 normal records in the evaluation database. Since the number of normal records was higher than the number of abnormal and borderline records, we have selected abnormal and borderline records from the dataset. The borderline and abnormal states in the dataset account for 55% and 45%, respectively. We assumed that the class labels were uniformly distributed and that it was possible to use the dataset. The dataset was used to calculate models using the classification method on the teenager database.

**Figure 3.**
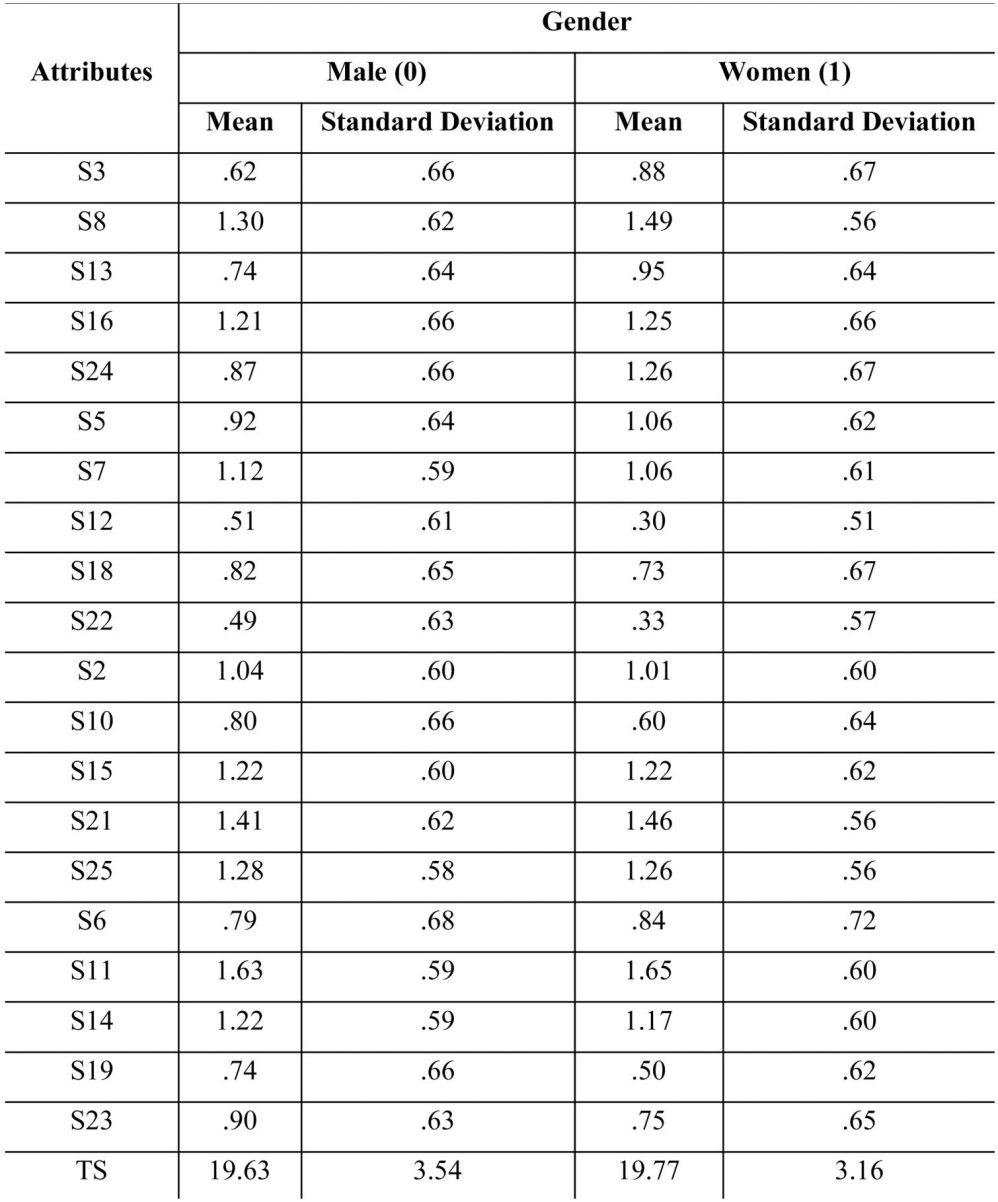
Descriptive statistics.

When studying the total score of the indicators depending on gender, 19.63 ± 3.54 for boys and 19.77 ± 3.16 for girls were not relevant. For each indicator, it is higher in men for indicators S12, S18, S22, S19, and S23, and higher in women for indicators S3, S8, S13, S24, and S5 (Figure 3).

Because the data collected using the SDQ is classified and the amount of data collected is large, a supervised learning method of machine learning, specifically a classification method of data mining, was considered appropriate. For the first time, machine learning methods were tested on adolescent emotional data, and supervised methods were deemed feasible.

We divided the database into 10 folds using cross-validation sampling and built models M1, M2, and M3 using the C5.0, bayes, and RIPPER algorithms, respectively. We evaluated the models using measures such as accuracy, sensitivity, and specificity. For Table 2, TP, TN, FP, FN tuples, and accuracy, sensitivity, and specificity measures in the confusion matrix for each model. The C5.0 model has a slightly higher accuracy of 0.865, sensitivity of 0.899, and specificity of 0.825, indicating that boundaries and abnormal states were predicted uniformly.

**Table 2.** Confusion matrix.

			Predicted		Accuracy	Sensitivity	Specificity
			1	2		1	2
**C50—M_1_**	Actually	1	49	7.9	0.866	0.899	0.825
		2	5.5	37.2			
**Naïve Bayes-M_2_**		1	2	8.8	0.856	0.898	0.805
		2	5.6	36.4			
**RIPPER-M_3_**		1	43	11	0.771	0.785	0.756
		2	11.8	34.1			
2-abnormal, 1-borderline							

The C5.0 and Bayesian models showed better results, so a *t*-test was used to determine whether there were statistical differences between the models (M_1_, M_2_). First, the error rates ${\;{\mathrm{err}(M}_{1})}_{i}$, ${\;{\mathrm{err}(M}_{2})}_{i}$ for each partition of the models were calculated, and then the mean error rates $\bar{\mathrm{err}}{(M}_{1}),\;\bar{\mathrm{err}}{(M}_{2})\;$were determined. The errors rates calculated for each partition were assumed to be k samples, the variance $\mathrm{var}(M_{1}-M_{2})\;$is calculated. A *t*-statistic with k−1 degrees of freedom was calculated for k samples. In our research work, k is 10. The significance *t*-test was used to determine the difference between M1, M2, was compared to the value of z = −2.26 from the distribution table corresponding to the 5%/2 significance level.

From Table 3, because we could not reject the null hypothesis, we conclude that any difference between M1 and M2 could be attributed to chance. Note: Bold values represent the final calculated test statistics for the Z-score confidence limit and the paired T-test, respectively

**Table 3.** Statistical tests of significance.

№	**C5**0${\;{\mathrm{err}(M}_{1})}_{i}$	$\mathrm{Bayes}{\;{\mathrm{err}(M}_{2})}_{i}$
**1**	0.11	0.17
**2**	0.11	0.14
**3**	0.13	0.17
**4**	0.18	0.15
**5**	0.15	0.15
**6**	0.12	0.13
**7**	0.12	0.10
**8**	0.15	0.14
**9**	0.12	0.16
**10**	0.14	0.12
$\underline{\mathrm{err}}$	0.13	0.14
$\underline{\mathrm{err}}{(M}_{1})-\underline{\mathrm{err}}(M_{2})$		0.0010
$\sqrt{var(M_{1}-M_{2})/k}$		0.009750648934
**Z confidence limit**		−2.26
***T* test**		−**0.94**

Bold values represent the final calculated test statistics for the Z-score confidence limit and the paired T-test, respectively.

**Figure 4.**
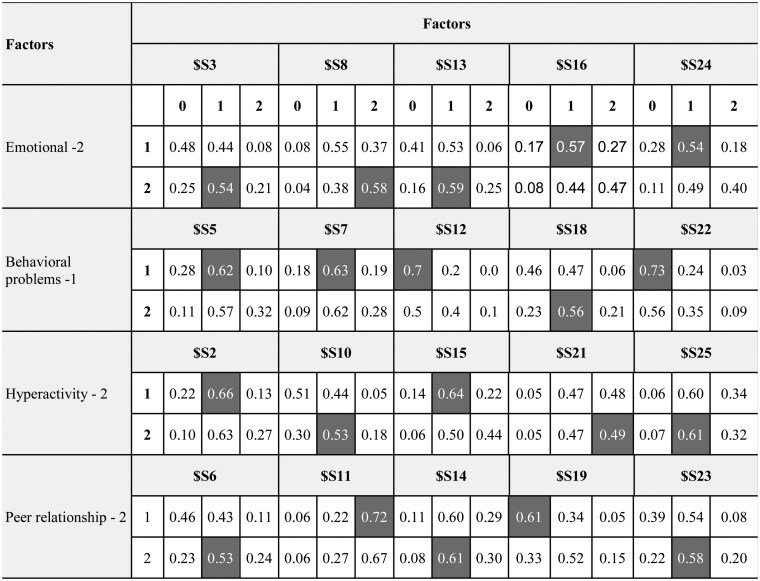
Bayes model.

The C5.0 model had slightly higher performance in terms of model performance evaluation measures. According to the *t*-test, there was no statistical difference between the models, and the C5.0 method resulted in trees with too many leaves, making them difficult to interpret. Therefore, in Figure 4, the results of the Bayesian model are shown.

For teenagers, the probability of developing emotional and behavioral disorders was about .57, mostly due to emotional factors such as often complaining of headaches (S3), worrying about many things (S8), and feeling unhappy (S13). However, due to emotional factors such as the tendency to be easily afraid of many things (S24) and to panic in new situations (S16), the probability of developing emotional and behavioral problems was .55.

Due to behavioral problems such as teenage often has temper tantrums (S5), generally obedient (S7), often fights with other children (S12), steals from home, school, or elsewhere (S22), the probability of the emotional and behavioral problems was 067. But depending on the indicator that they lies (S18), the probability of emotional and behavioral disorders was .56.

For hyperactivity, depending on factors such as restlessness (S2), easy distraction, and wandering concentration (S15), the probability of emotional and behavioral problems was .65. The disorders depend on some factors, especially constantly fidgeting (S10), thinking things out before acting (S21), and seeing tasks through to the end (S25).

For adolescents’ peer relationships, the probability of their emotional and behavioral problems was .66 and depends on the indicators, such as having at least one good friend (S11) and picking on or being bullied by other children (S19). However, disorders can occur due to factors such as playing alone (S6), not being liked by other children (S14), and getting on better with adults than with other children (S23).

## 4. Discussion

In a previous study, the emotional state of teenagers in Govi-Altai was examined using a database with 6 attributes. The class attribute represented their emotional boundary and abnormal type. Cross-validation was found to be more accurate than the holdout method, suggesting that it is more effective with larger datasets. The Bayesian model performed well in terms of accuracy (.890), sensitivity (.890), specificity (.890), and visibility.^17^

The study expands the teenager evaluation database by adding 21 attributes and employs cross-validation to create 10 folds for model building using classification methods. The C5.0 model achieved slightly better metrics in accuracy (.866), sensitivity (.899), and specificity (.899), but produced overly complex trees with many leaves. Statistical tests showed no significant differences between the models. Consequently, the Bayesian models was utilized for better interpretation of the results, focusing on visibility and ease of understanding. This is consistent with the strengths and limitations reported in international studies.

The first randomized controlled trial, conducted among 2301 children from Sukhbaatar District, Ulaanbaatar, the capital city of Mongolia, found that AOR for male children was 1.64 (1.29-2.10), AOR for low maternal education was 1.89 (1.16-3.05), AOR for low sleep was 1.40 (1.41-1.80), low physical activity AOR 1.31 (1.03-1.67), and prolonged screen time [AOR, 1.53 (1.20-1.94)] are associated with a higher risk of developing mental health problems.^18^ From 2013 to 2016 in Bulgan Aimag, Mongolia, mental health and risk factors were assessed using logistic regression with the Strengths and Difficulties Questionnaire (SDQ-Mongolia) from 1064 mothers of 6-year-old children. Overall, 9.5% of the children had diagnosed with emotional and behavioral scores (SDQ ≥17), and borderline scores (SDQ ≥14) together account for 20.1%. Maternal depressive symptoms (aOR, 1.66, 95% CI, 1.13-2.44), smoking by family members (aOR, 1.54, 95% CI, 1.06-2.21), and maternal alcohol consumption (aOR, 1.55, 95% CI, 1.02-2.33) were associated with higher rates.^19^ Within the framework of the “Healthy School Development” project, a survey was conducted among 4098 adolescents from September to November 2012-2013 in 4 low- and middle-income countries in Asia (Laos, Mongolia, Nepal, and Sri Lanka). Descriptive statistics, Chi-square tests, and logistic regression analysis were performed. Between 53.2% and 64.1% of the participants were female, and 33.7% in Sri Lanka and 53.8% in Laos were over the age of 15. Approximately 32.9% reported experiencing psychological distress, whereas 7.9%-13.2% reported suicidal ideation.^20^

A 1992 survey in Norway evaluated 11 369 adolescents from 67 schools, focusing on 10 832 participants aged 12-20, with a follow-up in 1994 involving 7425 participants. Researchers created a binary classification model using a Gradient Boosting Algorithm (GBM) to identify suicide attempts, training it on 70% of the sample and validating it with 10-fold cross-validation. The results indicated that 57.61% of suicide attempts were among high school students and 42.39% among junior high school students. The optimized GBM model achieved an AUPRC of 50.51% and an AUC of 88.58%, with a recommended cutoff of 0.0587, yielding a sensitivity of 0.854 and specificity of 0.758.^21^ A February 2021 survey of 400 high school students in Lagos found that 59.1% used alcohol, 23.6% used tobacco, 15.4% used cannabis, and 3.1% used cocaine. The performance of various classifiers—Decision Tree (DT), ZeroR, K-Nearest Neighbors (KNN), and Naive Bayes (NB)—was evaluated based on correctly classified instances (CCI). KNN outperformed the others, achieving the highest CCI rates for alcohol (82.40%), tobacco (66.22%), cannabis (91.16%), and cocaine (94.24%).^22^ This study used regression trees to analyze data from telephone surveys and found that peer smoking and alcohol consumption were significant predictors of adolescent smoking behaviors.^23^

Our survey on adolescent mental health in Mongolia uses data mining and machine learning to specify their mental health status, which is an advantage compared with previous studies. This work contributes to the field of health sciences in Mongolia and aligns with international research on adolescent mental health using similar techniques.

## 5 Conclusion

Comparing the model performance results with previous studies, Bayesian model accuracy decreased by .03, sensitivity decreased by .08, and specificity increased by .01. Additionally, the difference between the performance evaluation metrics of the C50 and Bayesian models is very small, between .01 and .02. This shows that the performance of the Bayesian method is good when the number of attributes in the database increases.

Compared to the knowledge generated by the research, the participants are more likely to develop emotional and behavioral disorders due to peer relationship indicators, such as other children generally not liking them and getting on better with adults, as well as emotional symptoms such as being unhappy and depressed.

In the future, factors affecting emotional and behavioral problems will be explored using advanced classification methods.

## References

[1] Bayarmaa V , Tuya N, Batzorig B, et al Using the strengths and difficulties questionnaire (SDQ) to screen for child mental health status in Mongolia. J Ment Disord Treat. 2017;3:2.

[2] Bayarmaa V , Nasantsengel L, Batzorig B, Chimedsuren O, Tuya N, Using the strengths and difficulties questionnaire (SDQ) to screen for children between 11 and 17 years old in a community sample. In: Asia-Pacific Psychiatry. Wiley-Blackwell; 2015.

[3] World Health Organization. *Mental Health of Adolescents* [Fact sheet]. World Health Organization; 2024. who.int

[4] Ministry of Health of Mongolia. *Adoption of Child and Adolescent Mental Health Care Delivery Scheme/Guideline* [Minister of Health Resolution]. Ministry of Health of Mongolia; 2021.

[5] Quinlan JR. C4.5: programs for Machine Learning. Elsevier; 2014.

[6] Pandya R , Pandya J. C5.0 algorithm to improved decision tree with feature selection and reduced error pruning. IJCA. 2015;117:18-21.

[7] Domingos P , Pazzani M. On the optimality of the simple Bayesian classifier under zero-one loss. Mach Learn. 1997;29:103-130.

[8] Rish I. An empirical study of the naive Bayes classifier. In: IJCAI 2001 Workshop on Empirical Methods in Artificial Intelligence. Seattle, WA: International Joint Conferences on Artificial Intelligence; 2001:41-46.

[9] Goodman R. The strengths and difficulties questionnaire: a research note. J Child Psychol Psychiatry. 1997;38:581-586. 9255702 10.1111/j.1469-7610.1997.tb01545.x

[10] Han J , Kamber M, Pei J. Data Mining Concepts and Techniques. 3rd edn. University of Illinois at Urbana-Champaign Micheline Kamber Jian Pei Simon Fraser University; 2012.

[11] Balamurugan M , Kannan S. Performance analysis of cart and C5. 0 using sampling techniques. In: 2016 IEEE International Conference on Advances in Computer Applications (ICACA). IEEE; 2016.

[12] Palmov SV , Miftakhova AA. Comparison of classification algorithms C4.5 and C5.0. Infokommunikacionnye Tehnologii. 2015;13:467-471.

[13] Yobero C. Determining Creditworthiness for Loan Applications Using C5. 0 Decision Trees. RPubs by RStudio; 2018.

[14] Benediktus N , Oetama RS. The decision tree C5.0 classification algorithm for predicting student academic performance. Ultimatics. 2020;12:14-19.

[15] Leung KM. Naive Bayesian Classifier. Department of Computer Science/Finance and Risk Engineering, Polytechnic University. 2007:123-156.

[16] Mining WID. Data Mining: Concepts and Techniques. Vol 10. Morgan Kaufinann; 2006:559-569.

[17] Tilyeubai A , Tsend J, Vaanchindorj B, et al Significance statistical test analysis on classification models of adolescent’s emotional problems. AAIML. 2023;03:1743-1757.

[18] Aoki A , Togoobaatar G, Tseveenjav A, et al Socioeconomic and lifestyle factors associated with mental health problems among Mongolian elementary school children. Soc Psychiatry Psychiatr Epidemiol. 2022;57:791-803. 34595562 10.1007/s00127-021-02178-7 PMC8483169

[19] Dagvadorj A , Corsi DJ, Sumya N, et al Prevalence and determinants of mental health problems among children in Mongolia: a population-based birth cohort. Glob Epidemiol. 2019;1:100011.

[20] Lee H , Lee EY, Greene B, et al Psychological distress among adolescents in Laos, Mongolia, Nepal, and Sri Lanka. Asian Nurs Res (Korean Soc Nurs Sci). 2019;13:147-153. 31003005 10.1016/j.anr.2019.04.001

[21] Haghish E , Laeng B, Czajkowski N. Are false positives in suicide classification models a risk group? Evidence for “true alarms” in a population-representative longitudinal study of Norwegian adolescents. Front Psychol. 2023;14:1216483. 37780152 10.3389/fpsyg.2023.1216483 PMC10540433

[22] Ajibade S-SM, Oyebode O, Dayupay J, Gido N, Tabuena A, Kilag O. Data classification technique for assessing drug use in adolescents in secondary education. *J Pharm Negat Results*. 2022;13:971-977.

[23] Davaasambuu S , Batbaatar S, Witte S, et al Suicidal plans and attempts among adolescents in Mongolia. Crisis. 2017;38:330-343. 28228061 10.1027/0227-5910/a000447

